# Dichloroacetate and Salinomycin as Therapeutic Agents in Cancer

**DOI:** 10.3390/medsci13020047

**Published:** 2025-04-23

**Authors:** Sunny Hunt, Anita Thyagarajan, Ravi P. Sahu

**Affiliations:** 1 Department of Chemistry and Biochemistry, Oberlin College, 173 W Lorain St, Oberlin, OH 44074, USA; shunt1@oberlin.edu; 2 Department of Pharmacology and Toxicology, Boonshoft School of Medicine, Dayton, OH 45435, USA; anita.thyagarajan@wright.edu

**Keywords:** non-small-cell lung cancer, drug repurposing, dichloroacetate, salinomycin, chemotherapy

## Abstract

Cancer is the second leading cause of mortality worldwide. Despite the available treatment options, a majority of cancer patients develop drug resistance, indicating the need for alternative approaches. Repurposed drugs, such as antiglycolytic and anti-microbial agents, have gained substantial attention as potential alternative strategies against different disease pathophysiologies, including lung cancer. To that end, multiple studies have suggested that the antiglycolytic dichloroacetate (DCA) and the antibiotic salinomycin (SAL) possess promising anticarcinogenic activity, attributed to their abilities to target the key metabolic enzymes, ion transport, and oncogenic signaling pathways involved in regulating cancer cell behavior, including cell survival and proliferation. We used the following searches and selection criteria. (1) Biosis and PubMed were used with the search terms dichloroacetate; salinomycin; dichloroacetate as an anticancer agent; salinomycin as an anticancer agent; dichloroacetate side effects; salinomycin side effects; salinomycin combination therapy; dichloroacetate combination therapy; and dichloroacetate or salinomycin in combination with other agents, including chemotherapy and tyrosine kinase inhibitors. (2) The exclusion criteria included not being related to the mechanisms of DCA and SAL or not focusing on their anticancer properties. (3) All the literature was sourced from peer-reviewed journals within a timeframe of 1989 to 2024. Importantly, experimental studies have demonstrated that both DCA and SAL exert promising anticarcinogenic properties, as well as having synergistic effects in combination with other therapeutic agents, against multiple cancer models. The goal of this review is to highlight the mechanistic workings and efficacy of DCA and SAL as monotherapies, and their combination with other therapeutic agents in various cancer models, with a major emphasis on non-small-cell lung cancer (NSCLC) treatment.

## 1. Introduction

Globally, the incidence of many common cancers is increasing, which has contributed to cancer becoming the second leading cause of mortality in the United States and worldwide [1]. The common hallmarks of cancer include deregulated cellular metabolism, sustained cell proliferation, and the development of drug resistance [2]. Among the various cancer types, lung cancer is the leading cause of cancer-related deaths and the most frequently diagnosed malignant tumor in males and females [3]. This review highlights the mechanistic workings and efficacy of the antiglycolytic dichloroacetate and the antibiotic salinomycin as monotherapies, and their combination with other therapeutic agents in various cancer models, with a major emphasis on lung cancer, particularly non-small-cell lung cancer (NSCLC) treatment.

The widespread proliferation of lung cancer can best be attributed to various social factors, such as air pollution and the addictive properties of nicotine-containing inhalants [3,4,5]. However, while these factors offer explanations for the comparatively large proportion of cancer-related deaths (22%), they do not address the high difference in mortality that patients are initially presented with; it is 37% higher among males (37.4 per 100,000 population) than females (27.3 per 100,000) [6]. To provide answers about these statistics, one must first discuss the multiple lung carcinoma subtypes that patients develop. Non-small-cell lung carcinoma (NSCLC) is the most diagnosed form of lung cancer [7,8]. Although it has a 5-year survival rate of 65% in localized stages, it quickly drops to 37% upon metastasis, and down to 9% for advanced forms of the disease [6,9]. Small-cell lung carcinoma (SCLC) has a much lower initial 5-year survival rate, starting at 37% and dropping to 3% in advanced metastatic stages. However, a higher proportion of patients are diagnosed with NSCLC, with some figures presenting it as high as 85% of lung malignancy cases [6]. Despite the seemingly bleak nature of these statistics, most forms of lung cancer, especially those that fall under NSCLC, are still treatable with traditional agents, such as platinum-based chemotherapy, and new approaches, such as immune checkpoint therapy (nivolumab + ipilimumab). However, there continue to be important challenges present in the treatment of NSCLC.

The common challenges associated with the available therapeutic agents for the treatment of various common cancers, including NSCLC, include drug resistance to molecular-targeted therapies and traditional cytotoxic agents [10]. For example, elevated levels of the C-Raf proto-oncogene serine/threonine-protein kinase (CRAF) protein can induce resistance to BRAF inhibitors by reducing drug bioavailability. In some patients, BRAF inhibitor resistance can be reversed with MEK inhibitor synergies, but the majority of NSCLC patients will experience disease progression within a year of starting treatment [10]. Other resistance mechanisms include MET gene amplification [11]. While customized, targeted treatments in response to NSCLC have clinically proven efficacy, patient outcomes are complicated by tumor heterogeneity that is increased by the onset of metastatic disease [12]. As a result, oncogenic and acquired resistance continue to create challenges in the treatment of NSCLC.

Alongside traditional chemotherapy side effects, mutations in oncogenes, such as the epidermal growth factor receptor (EGFR), lead to the activation of tyrosine kinase [13,14]. Tyrosine kinases have been shown to catalyze a number of cellular activities, including oncogenic cell proliferation/migration, by contributing to the upregulation of downstream signaling pathways by the phosphorylation of tyrosine residues [15,16]. These observations have resulted in the development of tyrosine kinase inhibitors (TKIs), such as erlotinib, gefitinib, and osimertinib, which are frequently part of NSCLC treatment regimens. While targeted molecular therapies, such as TKIs, represent important advancements in cancer pharmaceuticals and are associated with increased survival rates, many patients often develop acquired resistance to these therapies within a year of starting treatment [17,18].

Notably, drug resistance is linked to multiple different mechanisms, one of which is autophagy: the process by which cancer cells remove external molecules from inside their membranes. Epigenetic alteration and tumor heterogeneity are also significant contributors to resistance against targeted therapies, which make NSCLC particularly difficult to treat [15,16]. However, resistance mechanisms can be combated by using combination therapies with synergistic cytotoxic effects and repurposing clinically recognized drugs to broaden the amount of usable anticancer medications.

## 2. Repurposed Anticancer Agents

A noticeable proportion of potential anticancer agents have traditionally been used as metabolic regulators, such as metformin and dichloroacetate, both of which target aspects of glycolysis. Their efficacy in pre-clinical studies has been linked to their effect on cancer metabolism (the Warburg effect). The Warburg effect is the phenomenon of cancer cells relying primarily on glycolysis as opposed to oxidative phosphorylation [19,20,21,22]. Accelerated glycolysis promotes the inhibition of pro-apoptotic factors, such as caspase activity, as well as the increased synthesis of cell DNA and organelles [19,23]. Dichloroacetate (DCA), a medication that is primarily used to treat lactic acidosis, has a modulating effect on cancer metabolism. DCA alters the Warburg effect by stimulating pyruvate dehydrogenase (PDH), which causes the inhibition of pyruvate dehydrogenase kinase (PDK). This process causes a switch from glycolysis to oxidative phosphorylation in mitochondria. DCA has been shown to significantly reduce cell migration and proliferation, as well as to promote apoptosis [24,25].

In addition to antimetabolites, among other promising approaches, the ability of antibiotics to target bacteria seems to have overlapping mechanisms with their ability to target cancer stem cells (CSCs). Bactericidal medications function as anticancer drugs by disrupting the mitochondrial processes of CSCs, in the same way they act in prokaryotes [26]. This aspect is important because cancer tissue, particularly after metastasis, allows antibiotics to mitigate the heterogeneous nature of cancer tissue. CSCs that undergo survival selection are theorized to be, in part, responsible for not only metastasis but also recurrence, compromising further the anticancer treatments that prospective patients may receive. By disrupting the function of CSCs, antibiotics, such as salinomycin (SAL), are potentially able to promote mass-scale apoptosis, and improve survival outcomes by selectively targeting CSCs, which can allow for more aggressive treatment [27,28,29].

Compared to other novel therapies being developed, such as targeted molecular inhibitors and immunological synergies, studying drugs such as DCA and SAL can reveal how to broaden the scope of potential anticancer agents that can be used. DCA and SAL are both strong examples of why pre-existing pharmaceuticals can be repurposed for anticancer purposes. Metabolic modulators, such as DCA, target cancer metabolism [24,25]. They mediate disease progression through mitochondrial stress and often have significant synergistic potential through the same mechanisms. Metabolic modulators tend to be well tolerated and have long histories of chronic clinical use due to the prevalence of disorders, such as diabetes mellitus. SAL is a broad-spectrum anti-microbial that has a powerful targeted apoptotic effect on CSCs [26]. The effects of SAL indicate the potential efficacy of other commercially available anti-microbials, especially antiparasitic drugs with similar mechanisms. The insights into anticancer mechanisms that therapeutics like DCA and SAL provide will help generate a wider variety of pharmaceuticals that can be used to combat tumor cell proliferation, resistance, metastasis, and recurrence [24,25,26,27,28,29].

The overall objective of this review is to explore the anticancer properties of DCA and SAL as monotherapies and, when used in combination, as a potential therapeutic option for treating NSCLC. Notably, with its powerful metabolic effect, DCA is a potent synergist that can enhance the selective cytotoxic effects of SAL on CSCs.

## 3. Pharmacology of Dichloroacetate

Dichloroacetate (DCA), or dichloroacetic acid, is a metabolic modulator that is rapidly absorbed into the body by its salt ions and is taken up by cells via the monocarboxylate transport system. To understand the mechanisms of DCA, it is necessary to discuss the pyruvate dehydrogenase complex (PDC), a collection of enzymes found in the mitochondrial matrix. The PDC links cellular glycolysis to the tricarboxylic acid cycle (TCA), as well as to oxidative phosphorylation (OXPHOS). The PDC catalyzes the rate-limiting step in the aerobic oxidation of glucose, pyruvate, alanine, and lactate to Acetyl- CoA, as well as the initiation of OXPHOS. DCA stimulates PDC activation by inhibiting pyruvate dehydrogenase kinases (PDKs). DCA binds to the N-terminal domain of PDK, which prevents it from functioning as a catalyst, reducing the phosphorylation of pyruvate dehydrogenase E1 subunit 1 alpha (PDHE1α), thereby maintaining the PDC in its unphosphorylated form and its role in OXPHOS. As a result, the PDC can continue to aid in the consumption of glucose via OXPHOS [30,31,32].

DCA was originally investigated as an anti-diabetes drug because of its selective ability to lower blood glucose levels in vivo. PDK is upregulated in diabetes, which oxidizes glucose in the blood and peripheral tissues. However, in non-ketotic type 2 diabetes patients, DCA has also been shown to decrease the amount of lactate and alanine because of its ability to lock the PDC in its unphosphorylated form. The effect of DCA on lactate has long been documented because its most popular clinical application has been in the treatment of lactic acidosis. It is widely considered to be the most effective compound for the reduction in lactate [32].

## 4. DCA as an Anticancer Agent and Its Synergy with Other Therapeutic Agents

A solid tumor’s microenvironment is defined by its disorganized vasculature and prevalent hypoxic zones [33,34]. The lack of oxygen induces a metabolic shift to glycolysis, since the mitochondria can no longer rely on oxidative respiration. Hypoxia causes the selective proliferation of cancer cells with high glycolytic metabolisms since they can survive in spaces with limited oxygen. These neoplastic cells then depend on aerobic glycolysis to rapidly proliferate and invade the surrounding tissue. Due to the tumor heterogeneity in many of these cancer cells, glycolysis is transcriptionally upregulated by PDKs, resulting in the suppression of mitochondrial OXPHOS [35]. Under aerobic glycolysis, there is also an increase in the fermentation of glucose into lactate. Hypoxia-inducible factor 1 alpha (HIF1α) is the gene that encodes PDKs and most all glycolytic processes, inducing other adaptive responses that aid in malignant growth, and has also been shown to interact with oncogene c-Myc to enhance glycolysis [36,37]. Its expression is upregulated in cancer due to an accumulation of pyruvate and lactate. DCA has been shown to reduce c-Myc expression by degrading HIF1α through what is suspected to be a post-transcriptional regulation mechanism [38]. Lactate is involved in tumor survival and proliferation because mono-carboxylic transporter (MTC) proteins shuttle it across the cytoplasm where it is used as a tumor energy source. Lactate drives the process of angiogenesis even under normoxic conditions, and its concentration can be used as a prognostic marker. As discussed previously, DCA redirects mitochondrial metabolism away from glycolysis to OXPHOS by the inhibition of PDKs. The increased stimulation of the PDC catalyzes the rapid oxidation of glucose and lactate, which is associated with the increased expression of HIF1α. DCA increases reactive oxygen species (ROS), which induce downstream changes in mitochondrial function, causing the selective apoptosis of cancer cells. DCA also targets lactate through the same PDK inhibition mechanism [34,39,40,41,42,43].

The ability of DCA to prevent cancer cell proliferation has been directly observed in A549 and H1299 lung adenocarcinoma cell lines. In a study conducted by Allen and colleagues, it was found that treatment with DCA decreased glucose consumption and lactate production in vitro in a manner that was statistically significant compared to the controls [42]. DCA was also shown to greatly increase the A549 cell population doubling time. By directing tumor cells to use OXPHOS, DCA inhibits their ability to multiply and therefore proliferate [42]. While this study did not show that a DCA-induced metabolic shift was able to promote cell apoptosis or necrosis, it enhanced the sensitivity of A549 and H1299 cells to X-ray-induced cell killing. A summary of the in vitro and/or in vivo studies is given in Table 1.

Another anticancer property of DCA is its effect on autophagy, the homeostatic process by which cells degrade organelles and unusable proteins. This process is also a driving factor of acquired chemotherapy resistance [44,45]. Autophagy protects cancer cells by encapsulating the anticancer agents within a cell’s autophagosomes and degrading them with lysosomal enzymes [46,47,48]. DCA has been shown to induce autophagy instead of inhibiting it. In a study conducted by Lin and colleagues on colorectal cancer cell lines, it was determined that while DCA caused a G1 phase cycle arrest and reduced the growth of HCT116 WT, HT29, and PC3 cell lines, there was an overexpression of LC3B II, which is an indicator of autophagy. This was confirmed via electron microscopy, which revealed double-membrane autophagosomes in all the groups that underwent DCA administration [47]. The DCA-induced induction of autophagy was found to be mediated by the generation of ROS, the inhibition of the mammalian targets of rapamycin (mTOR), lactate excretion, the pyruvate-to-lactate exchange rate (k_PL_), and increased NAD+ and ratio of NAD+/NADH. In an in vivo study, DCA decreased the growth of HT29 tumor xenografts, which was mediated via reduced k_PL_ and increased autophagy [47]. Nevertheless, DCA has also been shown to inhibit autophagy in A549 and H1975 NSCLC cell lines [48]. By examining the essential autophagy proteins, LC3 and sequestosome (SQSTM; p62), after DCA administration, Lu and colleagues found that LC3 decreased while p62 levels increased, both of which are hallmarks of autophagy inhibition [48]. In vivo studies have demonstrated that DCA inhibits the growth of A549 and H1975 tumor xenografts and enhances the survival of tumor-bearing nude mice [48].

Despite its promising anticancer properties, environmental exposure to DCA has been reported to cause lung irritation, cough, shortness of breath, and bronchitis [49]. However, the most clinically limiting side effect of DCA is peripheral neuropathy. Chronic DCA exposure is suspected to cause oxidative stress in Schwann cells due to glycolytically mediated ROS generation. DCA-induced oxidative stress often results from reactive molecules, which form DNA/protein adducts from interference with amino acid catabolism and heme synthesis [50]. Alongside potentially degrading Schwann cells, DCA diminishes myelin-associated proteins, indicating that its effects on the nervous system could also be the result of demyelinating neurons [51]. Thus, the careful consideration of the optimal dose of DCA is needed to not only prevent its side effects but also to exploit its ability to improve the efficacy of other therapeutic agents.

Furthermore, DCA exerts synergistic potential with the most widely used chemotherapy agent, paclitaxel, on NSCLC cells. Studies by Lu and colleagues demonstrated that DCA enhanced the paclitaxel-induced inhibition of in vitro proliferation and autophagy of A549 and H1975 cell lines, as well as tumor growth and increased mice survival, indicating that DCA enhances chemotherapy efficacy [48]. Along similar lines, studies by Sun and colleagues have found that PDK2 inhibition via siRNA-induced downregulation was able to disrupt Warburg metabolism and resensitize paclitaxel-resistant A549-R cells to paclitaxel [52]. The combination of paclitaxel with the PDK2 inhibitor DCA resulted in the inhibition of the viability of A549-R cells in a synergistic manner. These findings indicate that the antiglycolysis mechanisms of DCA allow it to synergize with paclitaxel [52].

Platinum-based chemotherapies, such as cisplatin, are commonly used to treat metastatic NSCLC. DCA is a strong contender as an anticancer drug for NSCLC, in part because of its ability to synergize with cisplatin. Alongside its ability to shift the Warburg effect, DCA has been shown to synergistically inhibit cervical cancer (HeLa) cells in combination with cisplatin in vitro [53]. Xie and colleagues suggested that the unique metabolic action expressed by DCA, such as shifting HeLa cells’ metabolism from aerobic glycolysis to glucose oxidation, results in decreased mitochondrial membrane potential and increases the susceptibility of apoptosis-sensitized HeLa cells to the cytotoxic effects of cisplatin [53]. Moreover, Garon and colleagues determined the effects of DCA with or without cisplatin and docetaxel in NSCLC cell lines under normoxic and hypoxic conditions [54]. The data demonstrated the limited sensitivity of the DCA in all 54 tested NSCLC cell lines. Under hypoxic conditions, the DCA and cisplatin combination resulted in the inhibition of cell growth of a few cell lines (e.g., A427 and HCC-827). However, this combination did not show any synergistic effect on the SKLU-1 and A549 cell lines. In contrast, the DCA and docetaxel combination did not exert a synergistic effect [54]. In addition, a clinical trial conducted on seven patients suggested that oral DCA did not benefit previously treated NSCLC patients. The limitation of this study was the number of recruited patients. Thus, this warrants further studies to explore the efficacy of DCA as a sensitizing agent with combination therapy [54].

While most DCA anticancer mechanisms are involved in limiting growth and proliferation, they can also directly promote cell suicide. For instance, an apoptosis-promoting enzyme, caspase 3/7, is released upon high doses of DCA. These anti-proliferative effects are best attributed to the inhibition of pyruvate. Alongside its effects on glucose consumption, DCA was also observed to have an impact on angiogenesis, which ensures the supply of oxygen and nutrients to cancer cells [55]. This property was investigated using human umbilical vein endothelial cells (HUVECs) capillary structures [55]. In addition, DCA was found to enhance cisplatin chemotherapy, as well as gefitinib- or erlotinib-targeted therapy-induced decreased cell viability. Importantly, an in vivo study demonstrated the inhibitory effects of DCA on tumor xenograft growth in a chick embryo chorioallantoic membrane model, as well as on LNM35 tumor xenografts in nude mice [55]. The limitations of that study included the inability of DCA to inhibit in vitro cell migration and invasion, as well as the incidence/growth of lymph node metastasis in vivo [55]. Overall, the findings indicated that the efficacy of DCA is due to its ability to disrupt angiogenesis [55]. Thus, a reduction in angiogenesis is another anti-proliferative mechanism of DCA and is likely linked to its ability to reduce lactate concentration via PDK inhibition [56,57].

In addition to synergizing with cisplatin, DCA has also been shown to be a significant contender for combination therapy with TKIs, targeted therapeutics that have become a standard treatment for NSCLC [58]. Together with erlotinib or gefitinib, DCA has been shown to decrease the viability and cell proliferation of, and promote apoptosis in, EGFR-mutant, NCI-H1975, and NCI-H1650, but not in A549 or NCI-H460 NSCLC cell lines, which do not harbor EGFR mutations. In this study, Yang and colleagues provided compelling evidence that in EGFR-mutant (not EGFR wild-type) cell lines, erlotinib or gefitinib suppresses the activation of EGFR, along with AKT and extracellular signal-regulated kinase 1/2 (ERK1/2), two key proteins that are present in the downstream of EGFR signaling but have no effect on PDK activation. In contrast, DCA inhibited PDH activation in EGFR-mutant cells, but did not exert any effects on EGFR, AKT, or ERK1/2 phosphorylation [58]. On the other hand, in EGFR wild-type cells, erlotinib or gefitinib did not exert any effect on the activation of EGFR, AKT, or ERK1/2, whereas DCA inhibited PDH activation. Similarly, the combination of DCA with either erlotinib or gefitinib had pronounced effects on the inhibition of EGFR, AKT, and ERK1/2 phosphorylation, but did not affect PDH phosphorylation in EGFR-mutant cells or vice versa, indicating that DCA and EGFR TKIs exert independent functions in targeting signaling cascades [58].

Additionally, the anti-autophagic property of DCA has been shown to have a synergistic effect with another traditional chemotherapy agent, doxorubicin, which is commonly used to treat breast cancer [59]. Unfortunately, doxorubicin can be a potent autophagy inducer, including in the MDA-MB-231 breast cancer cell line. To that end, the authors of that study demonstrated that the previously known autophagy inhibition mechanisms exhibited by DCA were able to desensitize doxorubicin-resistant cells and enhance its cytotoxic effects [59]. This effect was further tested in vivo in mice, where DCA was shown to increase the anticancer effects of doxorubicin. These studies highlight the ability of DCA to synergize with traditional NSCLC agents, often sensitizing their cytotoxic capabilities and combating resistance mechanisms [59].

Another chemotherapy agent that DCA has been shown to be synergistic with is capecitabine. This combination is of particular interest because, like DCA, capecitabine has a high oral bioavailability. To that end, studies by Zheng and colleagues demonstrated that despite having little effect as a monotherapy, DCA significantly increased the antitumor effects of capecitabine in vivo in B-16 mouse allograft and A549 mouse xenograft models [60]. This effect was mediated by sensitizing apoptosis via remarkably increasing the cleavage of pro-caspases 8, 9, and 3, resulting in a reduced effective dose of capecitabine [60].

In addition, DCA has been shown to synergize with non-pharmacological cancer treatments as well. De Mey and colleagues investigated how DCA’s mechanisms can sensitize breast cancer cells to hypoxic radiotherapy treatment [61]. They hypothesized that under hypoxic conditions, pyruvate delivery into the mitochondria causes an upregulation of ROS levels, which radio-sensitizes cancer cells. This was partially confirmed by ROS-induced apoptosis and sensitivity in vivo [61]. Cook and colleagues showed how the metabolic actions of DCA affect high-grade glioma (HGG) cells [62]. Along with upregulating glycolysis, HGG cells are capable of utilizing glucose coupled with OXPHOS. HGG tissue is highly heterogeneous and OXPHOS is a contributor to chemo/radiotherapy resistance in this type of tumor. These factors put the efficacy of DCA as an anticancer agent into question. However, even at low concentrations, DCA was shown to radio-sensitize HGGs and induce apoptosis via ROS-induced DNA damage, cell cycle arrest, and an improved in vivo survival rate [62]. These effects imply that even in highly heterogeneous tissues, DCA still functions as a metabolic modulator.

DCA has also been shown to have anticancer synergies with various non-traditional agents, the most prominent of which is metformin. Metformin, like DCA, is a metabolic modulator that has a clinical history of being used to treat diabetes and hyperglycemia. As discussed in the introductory paragraphs, antimetabolites that interrupt glycolysis have strong potential as anticancer therapeutics because of their possible effects on Warburg metabolism. Li and colleagues found that DCA and metformin were able to synergistically inhibit the growth of and induce apoptosis in ovarian cancer cells [63]. Interestingly, it was found that while metformin sensitized DCA cytotoxicity via reducing DCA-induced anti-apoptotic Mcl-1 protein expression and protective autophagy, DCA sensitized metformin’s effects via decreasing metformin-induced glucose consumption and lactate accumulation [63]. The in vivo studies complemented the in vitro findings that DCA and metformin inhibit the growth of tumor xenografts in nude mice in a synergistic manner, indicating the promising efficacy of this combination against ovarian cancer [63]. Similar results were achieved by Haugrud and colleagues, who demonstrated that DCA and metformin’s combination synergistically induces cell death via caspase-dependent apoptosis in MCF7 and T47D human breast cancer cell lines [64]. Mechanistically, this effect involves oxidative damage via the inhibition of PDK1, with the simultaneous attenuation of metformin-induced lactate production [64]. These findings indicate that DCA and metformin’s combination could be explored as a promising therapy for breast cancer.

Metformin, however, is not the only non-traditional anticancer agent that DCA has been shown to be synergistic with. PX-478 is a small molecule that interferes with the transcription and translation of hypoxia-inducible factor-1α (HIF-1α) and leads to the diminished deubiquitinating of HIF-1α. Parczyk and colleagues determined that DCA and PX-478 have potential as a combination therapy for a variety of cancer cell lines (colorectal, lung, breast, cervical, liver, and brain) [65]. The suggested mechanisms include a strengthened inhibition of HIF-1α and increased ROS-mediated apoptosis of mitochondria through the targeting of PDKs. While DCA suppresses PDK-1, HIF-1α increases PDK-1 expression. Thus, PX-478 reinforces the primary effect of DCA indirectly, thereby synergistically increasing ROS production when combined with DCA [65].

**Figure 1:**
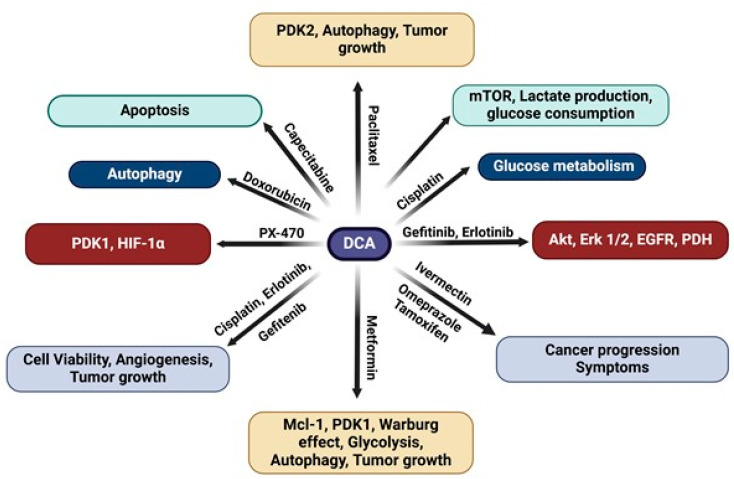
Schematic representation of mechanisms of action of DCA, both alone and its synergy in combination with other anticancer agent(s).

Another compound that DCA has been shown to have a strong synergism with is ivermectin. In a case report by Ishiguro and colleagues, DCA in combination with ivermectin, tamoxifen, and omeprazole was shown to relieve the symptoms of cancer progression [66]. Mechanistically, tamoxifen induces caspase-dependent cell growth inhibition and omeprazole enhances the antitumor effects of DCA, while ivermectin, an antiparasitic agent commonly used in veterinary medicine, exhibits growth-inhibitory effects on cancer cells via its ability to cause mitochondrial dysfunction and oxidative damage [66]. Given that cancer-related pain is a significant source of morbidity and decreases the quality of life of patients, by exhibiting the same antitumor mechanisms, these agents have been shown to relieve the advanced symptoms accompanying cancer progression, including pain [66]. A schematic representation of the synergistic mechanisms of DCA, both alone and with other anticancer agents, is shown in Figure 1. A summary of the in vitro and in vivo studies demonstrating the effects and mechanisms of DCA alone, as well as its synergy with other anticancer agents in various cancer models, is given in Table 1.

**Table 1:** Summary of in vitro and/or in vivo studies of DCA alone or its combination with other therapeutic agent(s).

Drug	Cell Line(s)	Target(s)	Key Findings	Refs.
DCA	A549, H1299	Lactate production/ glucose consumption	DCA decreases lactate production and glucose consumption.	[42]
DCA	HCT116 WT, HT29, PC3	Autophagy (LC3B ll protein), mTOR, lactate excretion, tumor growth	DCA induces autophagy in colorectal cell lines via inducing ROS generation and inhibiting mTOR and lactate excretion.	[47]
DCA, paclitaxel	A549, H1975	Autophagy (LC3 and p62 proteins), tumor growth	DCA inhibits autophagy, decreases cell proliferation and tumor growth, and increases mice survival.	[48]
DCA, paclitaxel	A549-R	PDK2	DCA resensitizes paclitaxel-resistant cells through PDK2 inhibition and siRNA-mediated downregulation.	[52]
DCA, cisplatin	HeLa	Glucose metabolism	DCA is able to shift glucose metabolism to sensitize HeLa cells and exert synergy with cisplatin.	[53]
DCA, cisplatin, decetaxel	54 NSCLC cells	-	Under hypoxic conditions, DCA and cisplatin (not docetaxel) combination enhances the sensitivity of a few NSCLC cell lines.	[54]
DCA + cisplatin/ gefitinib or erlotinib	A549, LNM35	Cell viability, angiogenesis, tumor growth	DCA decreases cell viability, disrupts angiogenesis, and reduces tumor growth.	[55]
DCA, gefitinib, erlotinib	NCI-H1975, NCI-H1650, A549, NCI-H460	EGFR, AKT, ERK1/2, PDH signaling	DCA in combination with gefitinib or erlotinib significantly inhibits the cell viability, promotes the apoptosis, and suppresses the activation of EGFR, AKT, and ERK1/2 signaling in EGFR-mutant NSCLC cell lines.	[58]
DCA, doxorubicin	MDA-MB-231	Autophagy	DCA sensitizes doxorubicin-induced cell death via autophagy inhibition, and its mechanism resensitizes doxorubicin-resistant cells and enhances the chemotherapeutic effect.	[59]
DCA, capecitabine	A549, B-16	Caspases/ apoptosis	DCA enhances the cytotoxic effects of capecitabine by promoting the release of caspases 3, 8, and 9.	[60]
DCA, metformin	SKOV3, OVCAR3	Mcl-1, Warburg effect, glycolysis, autophagy	DCA and metformin’s combination synergistically inhibits cell viability via inducing apoptosis, and reduces the growth of tumor xenografts.	[63]
DCA, metformin DCA, PX-478	MCF7, T47D MCF-7, MDA-MB-231, A549, H441, HEK-293, U251, HeLa, HEPG2, HT-29	PDK1, glycolysis HIF-1α, PDK1	DCA and metformin’s combination synergistically induces cell death and oxidative damage via PDK1 inhibition. DCA synergistically inhibits cancer cell growth with HIF-1α inhibitor PX-478 via inducing ROS-mediated apoptosis.	[64,65]
DCA, ivermectin, omeprazole, tamoxifen	Clinical trial	Cancer progression symptoms	DCA in combination with ivermectin, omeprazole, and tamoxifen relieves metastatic cancer-induced pain in patients.	[66]

*DCA*, dichloroacetate; *LC3*, microtubule-associated protein 1 light chain 3; *LC3B*, LC3 isoform B; p62, sequestosome-1; mTOR, mammalian target of rapamycin; *AKT*, Ak strain transforming protein kinase B; *ERK1/2*, extracellular signal-related protein–serine/threonine kinases 1 and 2; *PDK2*, pyruvate dehydrogenase kinase 2; *EGFR*, epidermal growth factor receptor; *HIF-1α*, hypoxia-inducible factor 1-alpha.

## 5. Pharmacology of Salinomycin

Salinomycin (SAL) is a polyether ionophore with an extended history of anti-microbial usage. It belongs to a selection of naturally occurring molecules comprising numerous cyclic ether groups [67,68]. Moreover, a characteristic feature of the SAL structure is the presence of a unique tricyclic 6-6-5 bis-spirochetal ring system with cis isomerism, which stiffens the whole compound [67,68]. It was isolated from Streptomyces albus, a species of bacterium, and produced by tank fermentation technology [69].

Ionophores are a subtype of anti-microbials that promote the transport of ions across the cell membrane. There are two different groups of ionophores: mobile carriers that bind ions to form lipid-soluble complexes and channel-forming compounds. Mobile carriers, such as SAL, are classified as carboxylic (neutral-charged) ionophores. By being a carboxylic transporter, it can ease the ion flux through to the cytoplasmic and mitochondrial membranes [69]. SAL and other antibiotics of its kind have been routinely added to the diets of poultry and livestock to prevent the spread of bacterial infections. However, SAL has been shown to be a broad-spectrum anti-microbial agent that can be used to treat more bacterial species. In addition, SAL is strongly antiparasitic, since it is used to effectively treat the spread of coccidiosis, a parasitic infection that can cause major economic loss in poultry farming, and has been shown to be able to eliminate malaria in vitro [70]. Alongside its antibacterial and antiparasitic uses, SAL has also been proven to possess properties that are anti-viral, antifungal, and even anti-inflammatory [70,71,72,73].

The most plausible explanation for this wide range of anti-microbial abilities lies within its proposed mechanisms. SAL has been shown to inhibit oxidative mitochondrial metabolism via ionophoric action. Via a complexation reaction, SAL disrupts the sodium/potassium ion balance across mitochondrial and cell membranes by the rapid efflux of K^+^. The ion reactions catalyzed by SAL lead to mass-scale apoptosis [74]. However, redirecting the flux of K^+^ is only one of SAL’s mechanisms that cause cell death. Notably, ferroptosis is a unique form of anti-cellular activity that is distinct from apoptosis and necrosis; it is instead dependent on autophagy. Autophagy causes the degradation of the iron storage protein, ferritin, which consequently releases more Fe^2+^, resulting in oxidative injury via a Fenton reaction. Another feature of ferroptosis is an overwhelming, iron-dependent accumulation of lethal lipids and ROS. SAL and its derivatives prevent the movement of iron from the lumen to the cytosol, triggering an iron-depletion reaction that is characterized by the rapid degradation of ferritin [75]. Ironomycin, a more potent derivative of SAL, has been found to physically accumulate in lysosomes, which disrupts their functioning by an excessive overloading of iron [76,77,78].

## 6. SAL as an Anticancer Agent and Its Synergy with Other Therapeutic Agents

Cancer stem cells (CSCs) are one of the major challenges in the treatment of recurrence and metastasis. CSCs make up 2–5% of cells within a tumor matrix and have a variety of unique properties, which include the ability to asymmetrically divide, differentiate, and self-renew, alongside an inherent resistance to therapy [79,80,81]. CSCs play a pivotal role in cancer metastasis and their increased presence is strongly correlated with poor patient outcomes [81,82]. By only targeting non-CSCs, traditional chemotherapy agents provide more space for CSCs to evolve into a more aggressive malignancy [83]. The challenge of treating CSCs makes it exigent to find an agent that can selectively target them. SAL has been shown to selectively target CSCs in vitro and in vivo, but its mode of action is not fully understood. However, the high anti-CSC bioactivity of this antibiotic is most likely linked to its ability to act as complex cations, which results in changes in the ion gradient and intracellular ph. Its proposed mechanisms, such as changes in the ion gradient, could result in injury to CSC mitochondria because they need an H^+^ ion gradient to generate enough ATP via OXPHOS, while changes in the pH could cause the denaturation of key proteins in mitochondrial function [83].

Using an active fluorescent SAL conjugate, it has also been shown that the molecular initiating event leading to a selective reduction in the proportion of CSCs after SAL treatment is strictly connected to a net outflux of Ca^2+^ cations from the endoplasmic reticulum (ER) because of the SAL-mediated alkali–metal ion transport across the ER membrane. A chemical library screened by Gupta and colleagues demonstrated that SAL can decrease the percentage of CD44-high/CD24-low breast CSCs by 20-fold [84]. While SAL has been shown to be effective against breast, colorectal, and circulatory cancers in these previous studies, the CSC targeting mechanism seen in SAL is not entirely understood [84,85,86,87,88,89]. Another proposed mode of action is connected to its effects on the Wnt/beta-catenin transduction pathway [89]. The Wnt oncogene and its signaling component, beta-catenin, was confirmed by Zhang and colleagues to be significantly transcribed in lung cancer cells, and SAL was one of the compounds used to inhibit the Wingless-related integration site pathway (Wnt) signaling in their study [90]. By suppressing Wnt through interfering with beta-catenin/CREB-binding protein transcriptional complexes, SAL was shown to be able to significantly reduce cancer cell stemness within lung cancer cell lines. Along similar lines, SAL was shown to suppress the stemness of liver cancer stem cells (LCSCs) enriched from MHCC97H cells via the inhibition of Wnt/beta-catenin signaling [91].

Eliminating CSCs is far from the only anticancer property that has been observed for SAL. SAL has also been shown to suppress the epithelial–mesenchymal transition (EMT) as well as transforming growth factors (TGFs). EMT is a process that is pivotal to metastasis. Epithelial-like cancer cells are changed, in part, by the transforming growth factor β (TGFβ) into mesenchymal-adjacent cells. This process promotes their invasion into other tissues. In a study conducted by Koeck and colleagues, SAL was shown to inhibit the EMT-inducing effect of TGFβ when used at the lower dose, while the higher dose further enhanced the mesenchymal phenotype in HCC4006 cells [92]. In A549 and HCC4006 cells, cell migration was decreased. In the A549 cells, however, it was noted that EMT markers, such as E-cadherin, were downregulated and vimentin was upregulated, leading the researchers to hypothesize that SAL may inhibit cell migration and influence the TGFβ-related EMT by interfering with different signaling pathways [92]. This was further confirmed by Hwang and colleagues when they discovered that SAL inhibits the TGF-β1-induced EMT through the AMPK/SIRT1 signaling pathways in both A549 and H460 cell lines [93]. Researchers proposed that these two proteins act synergistically with SAL to inhibit TGF-β1 [93].

SAL has been shown to have multiple apoptosis mechanisms. A study by Arafat and colleagues demonstrated that SAL inhibits the viability of A549 and LNM35 NSCLC cells in a dose- and time-dependent manner via inducing caspase 3/7-mediated apoptosis [94]. Moreover, decreased cell migration and invasion, as well as colony formation, were observed during SAL treatment. These effects were found to be mediated via an SAL-induced increased expression of the pro-apoptotic protein, NSAID-activated gene (NAG-1), a member of the TGF-β super-family, also known as macrophage inhibitory cytokine (MIC-1) and growth and differentiation factor-15 (GDF-15) [94]. Another apoptotic mechanism is the induction of intracellular ROS, which can best be attributed to its ionophoric action. Kim and colleagues demonstrated that SAL inhibits the viability of PC-3, LNCaP, and DU-145 prostate cancer cell lines in a dose- and time-dependent manner, with non-malignant RWPE-1 prostate cells being less sensitive [95]. Using PC-3 cells, it was observed that SAL triggers apoptosis by elevating intracellular ROS levels, leading to the translocation of Bax protein to the mitochondria, cytochrome c (Cytc) release, and the activation of caspase-3 [95]. Another prominent apoptosis mechanism is the induction of autophagy. Studies conducted by Li and colleagues confirmed that SAL induces autophagic flux in A549, Calu-1, and H157 NSCLC cell lines and proposed that this autophagy induction was due to endoplasmic reticulum (ER) stress [96]. SAL was observed to upregulate ER stress-related proteins in a time-/dose-dependent manner, which was found to be mediated via the ATF4-DDIT3/CHOP-TRIB3-AKT1-MTOR axis. However, the autophagic response caused by SAL has been shown to counteract its apoptotic effects [96]. This similar effect was also observed by Jangamreddy and colleagues alongside the mitophagy and hyperpolarization of mitochondria in prostate and breast carcinoma cell lines, and to a lesser extent in human normal dermal fibroblasts [97]. This effect was due to autophagy being a cell protective mechanism. However, autophagic flux can be counteracted by inhibiting agents, such as SAL, which have been shown to possess potential anti-resistance properties as well, alongside their ability to selectively target CSCs [98]. Studies by Fuchs and colleagues observed that SAL induced cell death in multiple apoptosis-resistant cancer cell lines, but not in normal healthy human cells [99]. This effect was more enhanced in human Molt-4 CD4+ T-cell leukemia cells.

Despite its promising anticancer properties, SAL causes several adverse effects, such as neurotoxicity [100]. SAL was shown to disrupt Na+ and Ca_2_+ homeostasis in root ganglia neurons. The ionophoric action of SAL then results in calpain/caspase-mediated cell death [100]. In in vivo studies, SAL administration has been shown to cause paralysis of the extremities and reproductive damage in mice [101]. The reproductive damage consisted of the disruption of spermatogenesis and the shrinking of testes. Alongside decreasing the rate of fertility in male mice, SAL also reduced the rate of pregnancy in female mice [101]. Thus, careful consideration of the optimal doses of SAL is needed to not only prevent its side effects but to also exploit its ability to improve the efficacy of other therapeutic agents.

In addition to its use as a monotherapy, SAL has also been shown to exert synergy with other therapeutics. In a study conducted by Xiao and colleagues, NSCLC cell lines were treated with SAL and metformin [86]. The findings indicated that not only did the combination of SAL and metformin inhibit cell viability, but upon microscopic examination, they revealed a substantial increase in cell death and a subsequent decrease in cell density. These effects were found to be mediated by the inhibition of AKT, ERK1/2, mTOR, and the downstream signaling cascade p70 s6K, regardless of the EGFR, KRAS, ALK, and LKB1 status of the cell lines. A particularly important technique in this study was the use of alveospheres that stimulated CSC-modulated tumor heterogeneity [86]. It was revealed that the exposure of alveospheres of various NSCLC cell types to the same concentrations of metformin turned out to be less effective than in 2D cultures, whereas co-exposure to SAL significantly enhanced metformin efficiency [86].

An additional oncogenic pathway that SAL disrupts is constitutively active AKT (AKT-CA), through the downregulation of thymidylate synthase (TS) [102]. Thymidylate synthase is a key enzyme in the pyrimidine salvage pathway, and its increased expression is positively associated with chemotherapy resistance [102]. According to Tung and colleagues, SAL was able to exert a combined cytotoxic effect with erlotinib by inhibiting TS through siRNA-mediated silencing and subsequent AKT inactivation [102]. Similar results were observed by Ko and colleagues, who showed that SAL could sensitize NSCLC cells to cisplatin [103]. Agents that have been reportedly desensitized due to the presence of TS include erlotinib and platinum-based therapies, such as cisplatin, both of which are used in the treatment of advanced NSCLC [103,104,105]. Along similar lines, a study by Michalak and colleagues generated cisplatin-resistant ovarian cancer cell lines that exhibited an elevated expression of the genes related to drug transporters [106]. In this study, the authors demonstrated that SAL, in combination with other cytostatic agents, such as 5-fluorouracil (5FU) and gemcitabine, was able to resensitize ovarian cancer cell lines to cisplatin [106].

**Figure 2:**
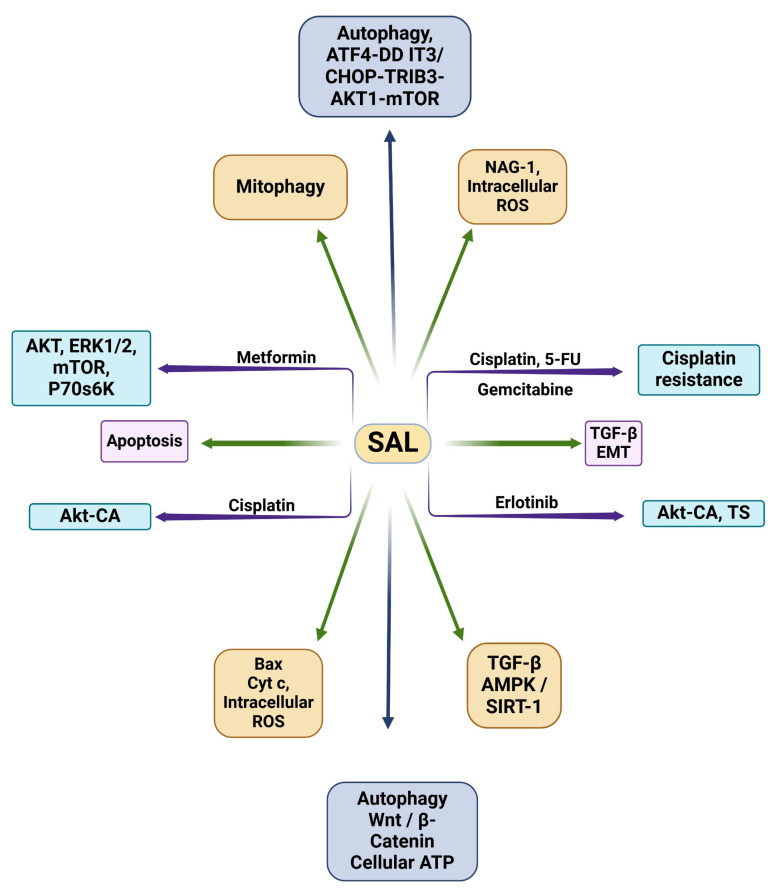
Schematic representation of mechanisms of action of SAL alone and its synergy in combination with other anticancer agent(s).

Moreover, SAL has also been shown to be a synergist with TKIs, such as gefitinib. A study by Zou and colleagues determined that SAL, in combination with gefitinib, was able to successfully induce apoptosis in gefitinib-resistant colorectal cancer cell lines regardless of their EGFR and KRAS status [107]. These effects were found to be mediated through the reduced activation of AKT, the loss of lysosomal membrane potential and mitochondrial membrane potential, and ROS production [107]. Additionally, it was also discovered that SAL and gefitinib overcame gefitinib resistance in Ras-overexpressing cells by the same mechanisms. Similar effects were also observed in vivo in mouse xenograft models, demonstrating that SAL and gefitinib’s combination sensitized gefitinib-resistant tumor xenografts to gefitinib [107]. A schematic representation of the mechanisms of SAL, alone and in combination with other agents, is shown in Figure 2. In addition, a summary of the in vitro studies on SAL, alone and its combination with other agents in various cancer models, is given in Table 2.

## 7. DCA and SAL as a Combination Therapy

Skeberdytė and colleagues were among the first to recognize that DCA had synergistic potential with SAL. In a study, the group tested the efficacy of DCA and SAL combination therapy on human colorectal cancer-derived cell lines DLD-1 and HCT116 [108]. DCA and SAL were found to synergistically inhibit the viability of the DLD-1 and HCT116 cell lines in 2D and 3D cultures. In both tests, DCA and SAL were reported to have a dramatically increased cytotoxic effect in combination, in contrast to their use as monotherapies. This was especially prevalent in the DLD-1 3D cultures, which had a greater prevalence of CSCs, supporting the previously discussed mechanisms observed for SAL [108]. In summary, the relationship between these two compounds seems to be the following: DCA sensitizes cancer cells via modulating the Warburg effect, making them more vulnerable to apoptotic agents, such as SAL [108]. This mechanism is viable because both DCA and SAL synergistically target mitochondrial function. Overall, DCA has been shown repeatedly to inhibit PDK in anaerobic glycolysis, while SAL redirects ion flux through the cytoplasm and mitochondrial membrane. Moreover, DCA has been shown, via a calcein fluorescence assay, to induce a greater cellular retention of SAL, which is another indicator of its ability to sensitize cancer cells [108]. One of the most concerning aspects of SAL is its frequently observed induction of autophagy in both combination and monotherapies. In that same assay, there was no observed calcein removal and the calcein fluorescence delay was shown to be mediated by DCA. This detail shows that DCA/SAL combination therapy has a lower susceptibility to resistance [108]. While the 3D cultures were able to imitate some of the complexity inherent in in vivo assays, more research is needed to validate these observations.

In their next studies on DCA and SAL, Skeberdytė and colleagues observed a similar effect in an LLC1 Lewis lung cancer cell line implanted in C57BL/6 mice [109]. DCA and SAL were found to significantly suppress tumor growth in vivo in the mice. In this report, there was a greater emphasis on studying the mechanisms of SAL on CSCs and the EMT process. In addition to preventing the local metastasis of LLC1 in C57BL/6 mice, DCA/SAL were shown to reduce the EMT transition and CSC markers in combination therapy. This is significant, because CSCs are not only an important player in metastasis formation, but also significantly contribute to the development of chemotherapeutic resistance and tumor relapse [109]. A few limitations of this study were that the majority of the generated results were performed with in vitro assays. Despite the promising findings, their applicability to complex systems remains limited [109]. Further studies should aim to investigate the effects of DCA and SAL combination therapy in vivo to properly elucidate their systemic effects on tumor heterogeneity and metastasis.

## 8. Conclusions and Future Perspectives

Metastasis and resistance continue to be some of the most important challenges in the treatment of malignancies, including NSCLC, indicating the need for alternative approaches. DCA and SAL have been shown to exert promising anticancer properties as monotherapies, and to also synergize with other therapeutic agents and regulate/inhibit cancer activities, including glucose metabolism, cell viability, and proliferation. Importantly, DCA/SAL combination therapy seems to have a particularly potent effect on the prevention of metastasis. SAL has been repeatedly shown to interrupt EMT and DCA enhances its cytotoxic effect, in addition to being an anti-proliferative agent. In regard to resistance, SAL selectively targets CSCs via mitochondrial dysfunction and DCA resensitizes cancer cells to apoptotic agents through metabolic modulation. This has been shown consistently by the cited research in this review, against multiple cancer types, including NSCLC cells. Autophagy, being one of the most significant potential caveats against recommending the usage of SAL, has been shown to be inhibited by DCA in NSCLC as well as in the colorectal cancer studies. Additionally, in the Lewis lung cancer study, a combination therapy was shown to have a much greater effect on the proliferation of CSCs and EMT markers in vivo.

While DCA and SAL’s synergy has not been exploited in great detail yet, their use as monotherapies has repeatedly been shown to be effective in in vitro and in vivo models of various forms of cancer, including NSCLC, with similar physiologies and accompanying challenges. In conjunction with chemotherapy and targeted molecular approaches, DCA and SAL combination treatment could be effective against resistance and metastasis in multiple tumor types, including NSCLC. In addition to DCA, its derivative, diisopropylamine dichloroacetate (DADA), has also been shown to modulate the tumor microenvironment (TME) of NSCLC via its ability to induce T-cell regeneration by decreasing lactic acid production [110]. Along similar lines, SAL-loaded liposomes containing tumor-derived nanovesicles have been shown to target the TME via inducing the immunogenic cell death of syngeneic colon cancer and melanoma tumor cells, mediated via enhanced antigen presentation by dendritic cells and macrophage polarization toward the M1 phenotype [111]. These studies have indicated that the use of DADA and SAL could be explored to enhance the efficacy of immunotherapeutic approaches for cancer treatment.

Notably, while the experimental results have indicated some anti-resistance efficacy of DCA and SAL, there are still more anti-resistance mechanisms to be elucidated. To that end, the utilization of the CRISPR/Cas9 system, an RNA-based adaptive immune system found in bacteria and archaea, has shown the potential to target the genes involved in cancer resistance [112]. The testing of agents like DCA and SAL through CRISPR/Cas9- isolated RNA could help researchers understand what resistance-contributing genes this synergy affects. Similarly, single-cell RNA sequencing could be used to understand how this synergy alters transcription factors and other mediators within metabolic and pro-apoptotic signaling cascades [113,114].

## Figures and Tables

**Table 2:** Summary of in vitro studies on SAL alone and its combination with other therapeutic agent(s).

Drug(s)	Cell Line(s)	Target(s)	Key Findings	Refs.
SAL	MHCC97H	Wnt/β-catenin	SAL suppresses the stemness of LCSCs via inhibiting Wnt/β-catenin signaling.	[91]
SAL	A549, HCC4006	TGFβ, EMT process	SAL interrupts EMT by inhibiting TGFβ signaling.	[92]
SAL	A549, H460	TGFβ, AMPK/SIRT1	SAL inhibits TGFβ through AMPK/SIRT1 signaling pathways.	[93]
SAL	A549, LNM35	NAG-1, intracellular ROS	SAL inhibits growth, migration, and invasion via inducing apoptosis through targeting NAG-1.	[94]
SAL	LNCaP, PC-3, DU-145, RWPE-1	Bax protein, cytochrome-c, intracellular ROS	SAL induces apoptosis by elevating intracellular ROS, cytochrome-c release to cytoplasm, Bax protein translocation to mitochondria, and caspase-3 substrate activation.	[95]
SAL	A549, Calu-1, H157	Autophagy, ATF4-DDIT3/CHOP-TRIB3-AKT1-mTOR	SAL induces apoptosis in lung cancer cells via autophagic flux.	[96]
SAL	PC-3, SKBR3, MDAMB468, MEF	Autophagy, mitophagy	SAL decreases cellular ARP, but induced autophagy can counteract its apoptotic mechanisms if not used in combination with autophagy inhibitors.	[97]
SAL	Molt-4 CD4+ T-cells	Apoptosis	SAL is able to induce apoptosis in apoptotic-resistant leukemia cells.	[98]
SAL, metformin	HCC4006, NCI-H1975, NCI-H2122, HCC95, NCI-H3122	AKT, ERK1/2, mTOR, p70 s6K	In combination with metformin, SAL induces cell death and decreases cell density/viability.	[86]
SAL, erlotinib	H1703, H1975	AKT-CA, TS	In combination with erlotinib, SAL inhibits TS and AKT signaling.	[102]
SAL, cisplatin	A549, H1703	AKT-CA	SAL and cisplatin synergistically inhibit lung cancer cells via AKT inhibition.	[103]
SAL, cisplatin, 5-fluorouracil, gemcitabine	A2780 CDDP, SK-OV-3 CDDP, A2780, SK-OV-3	Cisplatin resistance	SAL reverses cisplatin resistance in ovarian cancer cells in combination with 5-flurouracil and gemcitabine.	[106]
SAL, gefitinib	SW1116, LOVO, HCT-116, SW480, HT-29, NCM460	AKT	SAL and gefitinib’s combination induces apoptosis in gefitinib-resistant cell lines and overcomes gefitinib resistance in Ras-overexpressing cells.	[107]

*SAL*, salinomycin; *AMPK*, adenosine monophosphate-activated protein kinase; mTOR, mammalian target of rapamycin complex 1; *Wnt*, Wingless-related integration site pathway; *AKT-CA*, Ak strain transforming protein kinase B (Ca^2+^); *TS*, thymidylate synthase; *TGFβ*, transforming growth factor beta; *EMT*, epithelial–mesenchymal transition; *ROS*, reactive oxygen species; *SIRT1*, sirtuin gene 1; *NAG-1*, NSAID-activated gene; *ATF4*, activating transcription factor 4; *DDIT3/CHOP*, DNA damage-inducible transcript 3/C/EBP homologous protein; *TRIB3*, Tribbles pseudokinase 3; *AKT1*, AKT serine/threonine kinase 1.

## Data Availability

The data are included in this article.

## Abbreviations

NSCLC	Non-small-cell lung cancer
DCA	Dichloroacetate
SAL	Salinomycin
SCLC	Small-cell lung carcinoma
AMPK	Adenosine monophosphate-activated protein kinase
EGFR	Epidermal growth factor receptor
TKIs	Tyrosine kinase inhibitors
PDK	Pyruvate dehydrogenase kinase
CSCs	Cancer stem cells
PDC	Pyruvate dehydrogenase complex
TCA	Tricarboxylic acid cycle
OXPHOS	Oxidative phosphorylation
PDHE1α	Pyruvate dehydrogenase E1 subunit 1 alpha
HIF1α	Hypoxia-inducible factor 1 alpha
MTCs	Mono-carboxylic transporter proteins
ROS	Reactive oxygen species
HUVEC	Human umbilical vein endothelial cells
EMT	Epithelial–mesenchymal transition
TGFs	Transforming growth factors
TGFβ	Transforming growth factor β
Cytc	Cytochrome c
ER	Endoplasmic reticulum
HNC	Head and neck cancer
ERK1/2	Extracellular signal-regulated kinase ½
HGG	High-grade glioma
mTOR	Mammalian targets of rapamycin
LC3	Microtubule-associated protein 1 light chain 3
LC3B	LC3 isoform B
p62	Sequestosome-1
Wnt	Wingless-related integration site pathway
AKT-CA	Ak strain transforming protein kinase B (Ca^2+^)
TS	Thymidylate synthase
SIRT1	Sirtuin gene 1
NAG-1	NSAID-activated gene
ATF4	Activating transcription factor 4
DDIT3	DNA damage-inducible transcript 3
CHOP	C/EBP homologous protein
TRIB3	Tribbles pseudokinase 3
AKT1	AKT serine/threonine kinase 1
DADA	Diisopropylamine dichloroacetate
TME	Tumor microenvironment
